# Political economy analysis of the performance‐based financing programme in Afghanistan

**DOI:** 10.1186/s41256-021-00191-6

**Published:** 2021-03-10

**Authors:** Ahmad Shah Salehi, Karl Blanchet, Anna Vassall, Josephine Borghi

**Affiliations:** 1 London School of Hygiene and Tropical Medicine, Faculty of Public Health and Policy, Department of Global Health and Development, London, UK; 2 CERAH, University of Geneva, Geneva, Switzerland

## Abstract

**Background:**

Performance-based financing (PBF) has attracted considerable attention in recent years in low and middle-income countries. Afghanistan’s Ministry of Public Health (MoPH) implemented a PBF programme between 2010 and 2015 to strengthen the utilisation of maternal and child health services in primary health facilities. This study aimed to examine the political economy factors influencing the adoption, design and implementation of the PBF programme in Afghanistan.

**Methods:**

Retrospective qualitative research methods were employed using semi structured interviews as well as a desk review of programme and policy documents. Key informants were selected purposively from the national level (*n* = 9), from the province level (*n* = 6) and the facility level (*n* = 15). Data analysis was inductive as well as deductive and guided by a political economy analysis framework to explore the factors that influenced the adoption and design of the PBF programme. Thematic content analysis was used to analyse the data.

**Results:**

The global policy context, and implementation experience in other LMIC, shaped PBF and its introduction in Afghanistan. The MoPH saw PBF as a promise of additional resources needed to rebuild the country’s health system after a period of conflict. The MoPH support for PBF was also linked to their past positive experience of performance-based contracting. Power dynamics and interactions between PBF programme actors also shaped the policy process. The PBF programme established a centralised management structure which strengthened MoPH and donor ability to manage the programme, but overlooked key stakeholders, such as provincial health offices and non-state providers. However, MoPH had limited input in policy design, resulting in a design which was not well tailored to the national setting.

**Conclusions:**

This study shows that PBF programmes need to be designed and adapted according to the local context, involving all relevant actors in the policy cycle. Future studies should focus on conducting empirical research to not only understand the multiple effects of PBF programmes on the performance of health systems but also the main political economy dynamics that influence the PBF programmes in different stages of the policy process.

## Introduction

Performance-based financing (PBF) has become a popular financing mechanism in low and-middle income countries (LMICs) in the past 15 years [1]. PBF is defined as a cash payment issued after attaining and verifying predefined results [2]. PBF in the health sector comprises direct payments to health professionals such as doctors, nurses, and community health workers [3–6], organisations such as health facilities or medical groups [7], and government or non-government entities, typically based on quality and/or utilisation outcomes [8]. Those paying can be governments, donors, or insurance programmes [9]. PBF is seen not only as a tool to increase the motivation of healthcare workers and improve health systems performance but also a strategic purchasing reform [10]. PBF aims to improve outcomes by motivating healthcare workers through incentives [11], introducing a results-based culture where ‘doing business as usual’ is no longer the norm [12]. The introduction of PBF is supposed to generate a competitive environment which will motivate healthcare organisations to exhibit enhanced efficiency, high-quality services and improved results [13–16]. PBF is expected to change governance arrangements, strengthening relationships between levels of the health system, and improving regulation of the health sector and health financing [17–21].

Political economy analysis (PEA), which studies power and resource distribution and contestation, the roles played by different actors and their interactions, and how this shapes programmes and policies [22–24], is well suited to the study of PBF. While PBF can influence the behaviour of healthcare professionals, it can also affect the behaviour of other relevant actors and their relationships between each other [25]. Due to its innovative nature, PBF enforces distinct arrangements for the sharing of resources; and represents a risk or opportunity to actors as a result of changes to their roles and responsibilities and the modification of organisational processes [26]. Consequently, a new political and economic environment comes into existence. Nevertheless, minimal information is available regarding the political processes and interactions associated with PBF in addition to the factors that influence the choice and application of such policies. To date, only a limited number of PEA have been conducted on PBF programmes in low-income settings and fragile and conflict-affect states (FCAS) [27–31]. These studies partially examined political economy factors underpinning the adoption and implementation of PBF [29, 31], PBF policy processes [30], interaction between structure (historical legacies, context, institutions) and agency (agendas, actors, power relationships) concerning the implementation of incentive-based policies [27], and interplay between actors in formulating and implementing PBF programmes [28]. To the best of our knowledge, there is only one study concentrated on the political economy of PBF in a comprehensive manner from a low-income setting [31]. There is no study from FCAS. This justifies the need for a comprehensive application of PEA for PBF in low-income settings, especially FCAS. A PEA approach was used to understand the factors (context, actors, processes) influencing the PBF adoption, design and implementation in Afghanistan, and examine why the PBF programme in Afghanistan did not have intended effects.

To strengthen maternal and child health services, the Ministry of Public Health of Afghanistan (MoPH) with financial support from the World Bank (WB) implemented a PBF programme between 2010 and 2015 [32]. This programme provided incentives to healthcare workers to achieve improved coverage of essential maternal and child health services [33]. The programme had some effects on the utilisation and quality of health services; however, these changes were not statistically significant [34], and the programme was not cost-effective [35]. Therefore, the purpose of this study was to examine the political economy factors influencing the adoption, design and implementation of the PBF programme in Afghanistan.

## Methods

### Study Setting

In 2003, Afghanistan introduced the Basic Package of Health Services (BPHS) to ensure equitable access to a core set of health services in remote and underserved populations [36]. The BPHS has been contracted out by non-state providers (NSPs) in 31 of its 34 provinces while the BPHS was provided by the direct implementation of MoPH known as the “Ministry of Public Health Strengthening Mechanism” (MoPH-SM) in three provinces [37]. Under the MoPH-SM arrangement, provincial health offices were contracted by the central MoPH to provide BPHS services in those provinces [38].

The introduction of these reforms saw a substantial reduction in under-five and infant mortality rates from 257 to 165 per 1000 live births in 2001 to 97 and 76 per 1000 live births in 2010, and maternal mortality also declined substantially from 1600 to 2002 [39] to 327 per 100,000 live births in 2010 [40]. However, maternal and child mortality remain high compared at the regional level.

**Fig. 1:**
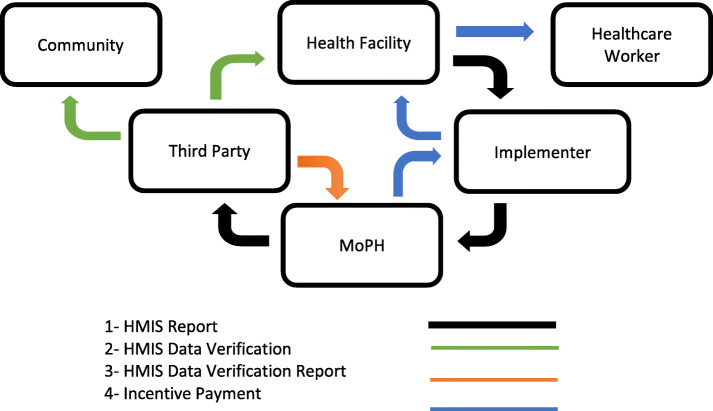
Afghanistan PBF programme arrangements

The PBF programme was initiated in 2010 in the context of BPHS to improve maternal and child health. In total, 463 health facilities in 11 out of 34 provinces were included in the programme. The PBF programme targeted the following maternal and child health services: antenatal care, delivery by skilled birth attendant, postnatal care, and pentavalent vaccination. Health workers were provided incentives based on extra production of outputs (targeted services) above the baseline reported by health information management system (HMIS) and verified by a third party (Fig. 1). Verification of the HMIS data occurred on a regular basis on a random selection of PBF health facilities and households. The PBF programme was evaluated by means of two household surveys: a baseline survey in 2010 and an end-line survey in 2015. Households living within the catchment area of a facility exposed to PBF were interviewed together with those living in the catchment area of control health facilities [32].

### Conceptual framework

A conceptual framework based on Buse et al. [23] was adapted to guide our data collection and analysis. Our framework helps understand the fundamental dynamics that influenced the PBF programme adoption, design and implementation. Our framework characteristics are as follows:

1. *Context*: Understanding the contextual factors such as social, economic and political setting as well as global factors which influence the adoption of PBF programme in Afghanistan.
2. *Actors*: Identifying the role, power, interest, and ideas of actors in relation to PBF and the extent to which they were involved in and affected the adoption, design and implementation of PBF. *Power* is considered to be the capability of agents to accomplish results in social practices [41], whether they are competing against each other or acting collaboratively. Power is acknowledged to be significantly influential on the process of developing and implementing policies [42]. *Interest* is considered to be the desire to do a particular thing. Those who are capable of influencing policy do this with the intention of enhancing their political and or economic interests. Actors who are not in government could have a specific interest in economic outcomes, whereas government actors’ interest might be driven not only by their personal economic interest but also their political interests, particularly in terms of sustaining their hold on power. *Idea* is consistently a key driver of policy, along with direct political or economic concerns. In situations where people can not rationally decide, idea provides directions in terms of the actions they should take to ensure consistency with their fundamental values and beliefs in life [43].
3. *Process*: The official PBF programme design and how it was implemented in practice, including nonconformities to the initial design and reasons for these.

**Fig. 2:**
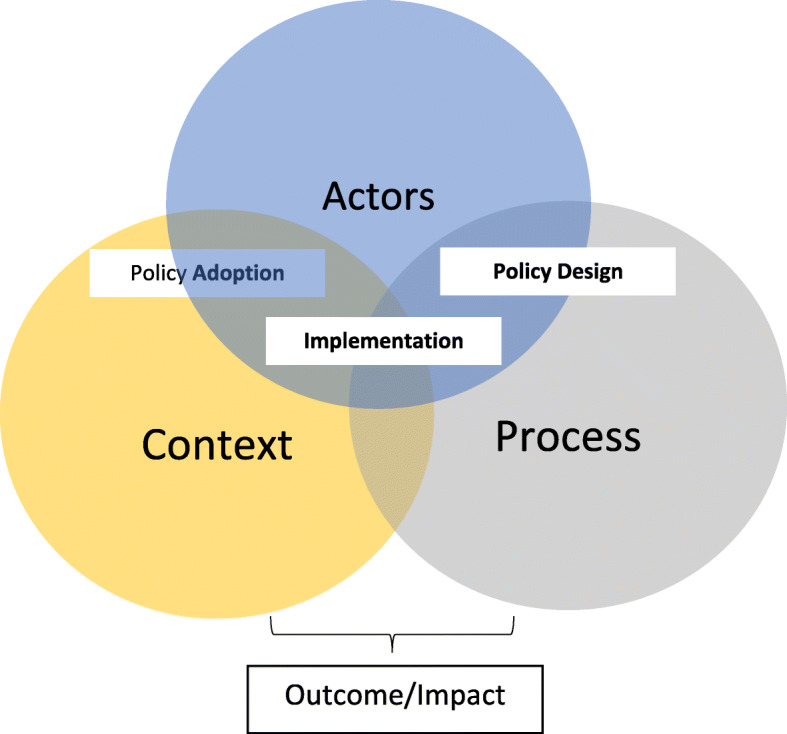
The study conceptual framework

Figure 2 presents the conceptual framework components and the interactions between actors and context in the adoption stage, between actors and process in the design and implementation stage. The framework takes the position that the dynamics between actors and the context in which PBF came into existence (adoption) and the process through which PBF programme was designed and implemented had influenced the performance of the PBF programme and subsequently the results.

### Study Sampling

Key informants, who have especially informed viewpoints on the PBF programme, were selected purposively from each level of the health system. At the national level, respondents were interviewed from the MoPH who managed the PBF programme, the World Bank who funded the PBF programme, the third party who conducted the PBF programme monitoring and data verification, and non-state providers (NSPs) who implemented the PBF programme in the BPHS health facilities. At the province level, health managers (HM) who were supervising the implementation of PBF programme were interviewed. At the facility level, healthcare workers (HW) who were providing healthcare services at health facilities were interviewed. Two provinces (Takhar and Balkh) were selected based on variations in population ethnicity and health facility geographical location. In total, 15 public primary care health facilities were selected that comprised urban (*n* = 5), semi-urban (*n* = 5), and rural health facilities (*n* = 5). In total, we interviewed 30 key informants, from national level (*n* = 9), from province level (*n* = 6) and facility level (*n* = 15) (Table 1).

**Table 1:** Research participants

Institution	Interviewee	Number	Reason for Selection	
MoPH	Deputy Minister of Policy and Planning	1	Led the negotiation process between MoPH and the donor when deciding on implementing a PBF programme	PM
	GCMU Team Member	2	Managed PBF procurement and financial management	PM
	HEFD Team Member	2	Coordinated and supervised PBF implementation	PM
	Provincial Managers	2	Provided key information about the context, content, and implementation of PBF for the respective provinces	HM
	PBF HMIS Team Member	1	Managed PBF reported data from the NSPs on a quarterly basis.	HM
Donor	Team Member	1	Represented the role and opinions of the donor supporting the PBF programme	PM
Third Party	Team Member	2	Verified the HMIS data and assessed the performance of PBF in BPHS health facilities by applying BSC and conducting household surveys	HM
Implementer (NSP)	Provincial Managers	4	Implemented the PBF, monitored implementation, understood the context and content of the programme	HM
	Heads of Health Facilities	4	Views of frontline managers on PBF implementation, its strengths and challenges, their satisfaction with PBF, and contextual factors	HW
	Healthcare Workers	11	Views of frontline workers on PBF implementation, its strengths and challenges, contextual factors, and their satisfaction with PBF.	HW

*MoPH* Ministry of Public Health; *GCMU* Grant and Contract Management Unit: *HEFD* Health Economics and Financing Directorate; *HMIS* Health Management and Information System; *NSP* non-state provider; *PBF* Performance-Based Financing; *PM* Policymaker; *HM* Health Manager; *HW* Healthcare Worker

### Data Collection

This study adopted retrospective qualitative research methods and conducted a review of documents related to Afghanistan’s PBF programme.

Qualitative interviews were designed with semi-structured questions and probes and were conducted in participants’ offices and health facilities over the phone by the principal investigator (PI). Interviews with the third-party evaluation organisation who conducted the PBF programme monitoring and data verification and donors were conducted in English and the rest were conducted in the local languages. Where respondents consented, a digital recording device was used to record interviews (*n* = 24), while notes were taken in six out of 30 interviews. All recorded interviews were transcribed verbatim by the PI. The research framework guided the questions, which focused on three major areas – the PBF programme context, actors and implementation process.

For the document review, the PI reviewed minutes of PBF coordination meetings and workshops, monitoring visit reports, PBF progress reports, donor mission reports (aide memoire), health facility and household survey reports from the impact evaluation, and published literature on Afghanistan’s PBF scheme.

### Data analysis

The data analysis was inductive as well as deductive, and it was following the objective of the study and our conceptual framework. ‘Content analysis’ was used to analyse the data [44]. First, all transcriptions and notes were carefully reviewed. Key themes were highlighted from the conceptual framework. Based on their relationships, data were selected and accommodated under specific thematic classes. Information on the same opinion was combined, and quotes were copied under the relevant classifications. Finally, each classification was studied and interpreted carefully. Common viewpoints between key informants were then described and important responses elucidated. A similar approach was used to incorporate the concerned content of reviewed documents under the related thematic classes. The findings from interviews were triangulated with other data sources (PBF document review, published literature review).

## Results

The respondents reported an age range from 26 to 59 years. All of them were married. Men and women comprised 60 % and 40 %, respectively, with education background from undergraduate to doctoral degree and experiences from 4 years to 35 years.

### PBF Programme Context

There was a range of contextual factors contributing to the introduction of the PBF programme in Afghanistan. First, at the global level, focusing on ‘results’ is a fundamental ideological shift from input-based financing to outputs and outcomes. PBF was regarded as an innovative solution to help utilize limited resources effectively and efficiently [21], and make progress towards global health goals: initially MDGs 4 and 5 [45], and subsequently Universal Health Coverage (UHC) [46]. Therefore, an increasing number of developing countries were adopting PBF schemes, and it was seen by local stakeholders to be desirable to join this global movement.

*“The funding trend at the global level was towards PBF programmes and Afghanistan could not miss this opportunity” [PM, National level).*

Second, at the local level, maternal mortality ratio was considered one of the highest worldwide at 1600 per 100,000 live births [39], contraceptive use was at 15 per cent, ANC use was at 36 per cent, and full immunisation was only at 37 per cent [47]. The World Bank first advocated for the idea of a PBF programme in Afghanistan, based on the positive experience of improving maternal and child health outcomes in Rwanda using PBF.

*“PBF was not a recognized term in the Ministry [MoPH]. It was the World Bank who attracted the attention of the Ministry towards PBF” [HM, national level]*.

The MoPH was also very receptive to the idea of PBF because of the experience of providing BPHS services through NSPs [48] using performance-based contracting (PBC) in which project payments to NSPs were subject to satisfactory performance of NSPs on a yearly basis [49]. The MoPH found the idea of PBF in line with the MoPH position and idea to be the steward of the health sector in Afghanistan and allow NSPs to implement the basic health services on behalf of MoPH (Ministry of Public Health 2005). Furthermore, PBF, involved the offer of additional financial resources to the health sector, just prior to presidential and parliamentary elections scheduled in August 2009 and December 2010. The fact that PBF offered the means to deploy more resources to mainly rural areas during an election campaign was also of paramount importance.

In a meeting held in November 2008, the Minister of Health confirmed his decisive support for the adoption of PBF. The MoPH expected that the PBF could expand maternal and child services and strengthen health systems.

*“The introduction of PBF is in a critical time when the country is going through some political and security turmoil; therefore, the announcement of the new funding for improving mothers and children health is considered as good news for people.”* (Ministry of Public Health 2008a).

### PBF Programme Actors

The key actors associated with the PBF programme were the central MoPH, Ministry of Finance (MoF), the WB, other donors, Provincial Health Offices (PHOs), non-state providers (NSPs), third party, healthcare workers including community health workers, and patients/clients. In this section, the roles of actors and how they influenced the design and implementation of PBF programme is presented. Table 2 presents the roles of PBF programme actors, and Table 3 presents the PBF programme actors’ matrix.

The MoPH showed interest in PBF and undertook numerous roles in adopting and managing the PBF programme. The major entities in the MoPH pertaining to the PBF programme were the Health Economics and Financing Directorate (HEFD) which was in charge of the overall management of the PBF programme; the Grant and Contract Management Unit (GCMU) which assumed responsibility for managing the PBF contracts and disbursing performance payments to implementers; the Health Information Management Information Unit (HMIS) which was responsible for the PBF programme technical reporting; the Monitoring and Evaluation Unit (M&E) which assumed responsibility for monitoring the PBF programme; and the PHOs which were in charge of routine monitoring and provincial level coordination of the PBF programme.

In our study, the HEFD emerged as a key actor among the MoPH entities in the context of PBF programme. The HEFD had established a close relationship with the MoPH central entities, MoPH PHOs, MoF, the third party and NSPs, and it served as the first contact point for coordination with the WB. The PBF National Coordinator who was placed in the HEFD was managing the PBF contracts with NSPs and third party in close coordination with the GCMU. Meanwhile, the PBF project placed two M&E national consultants, one HMIS national consultant and one financial management national consultant in the HEFD. The M&E consultants were reporting to the Coordinator while the HMIS and financial management consultants were reporting not only to the Coordinator but also to HMIS and financial management units to ensure the main units of the MoPH were closely linked to the PBF programme. The MoPH M&E unit was expected to assist the PBF programme with monitoring activities. Nevertheless, the function of the M&E unit was generally limited because the HEFD M&E national consultants undertook monitoring visits to the PBF health facilities [HM, national level].

**Table 2:** PBF programme key actors’ role

Actors		Roles
**MoPH**	**HEFD**	The MoPH HEFD assumed responsibility for the daily implementation of the PBF programme including monitoring and preparing yearly reports to track progress on programme implementation. In addition, HEFD cooperated with HMIS in organising training sessions for managers involved in PBF
	**GCMU**	GCMU assumed responsibility for processing and managing contracts for NSPs and for third-party organisations. The GCMU finance section assumed responsibility for conducting the financial management of the programme such as preparation of payment orders, fund disbursement, reports, and expenditure statements.
	**HMIS**	The MoPH HMIS Unit introduced changes to the HMIS data capture forms to enable its use to monitor PBF. Furthermore, they led training sessions for the implementers and PHO staff on the new HMIS, NMC, and other PBF-related events. The HMIS also had to maintain and supply any PBF-related HMIS information and provide reports on the main PBF indicators.
	**PHO**	The MoPH PHOs assumed responsibility for ensuring that oversight from the BPHS health facilities was conducted in coordination with the NSPs. Moreover, the PHOs were responsible for arranging provincial PHCC meetings.
	**M&E**	The MoPH M&E Unit assumed responsibility for managing and processing NMC data. The staff of M&E assisted the HEFD with monitoring activities associated with the PBF.
**MoF**		The MoF was the prime recipient of the PBF fund. The MoF role was to strengthen donor coordination, to ensure accountability and transparency, and to align donor funding in accordance with the country development objectives. The MoF delegated full authority in terms of technical decisions and project management to MoPH regarding PBF.
**The WB**		The WB provided financing assistance to PBF programme and played an operational role in appraising and monitoring PBF programme activities. The WB provided the final approval on the PBF procurement and financial plan, process of contracting NSPs and third party, the release of funds to implementers, hiring of staff, and adaptation of the design of PBF programme.
**Third Party**		The function of the third party was to verify HMIS data and conduct baseline and-end line surveys to evaluate the effect of the programme. Moreover, the third party had the responsibility for assessing the quality of PBF health facilities.
**NSP**		The NSPs assumed responsibility for implementing the PBF programme in the BPHS health facilities. They were expected to ensure the availability of quality health services to the people whom they were serving in accordance with their PBF BPHS contracts, as well as make an accurate record of any unintended effect of PBF on the delivery of health services.
**HW**		The healthcare workers provided health care services to people.
**Patient**		Patients were the prime beneficiary of health care services provided by healthcare workers.

*MoPH* Ministry of Public Health; *HEFD *Health Economics and Financing Directorate; *GCMU* Grant and Contract Management Unit; *HMIS* Health Management and Information System; *HW* Health Worker; *PHO* Provincial Health Office; *M&E* Monitoring and Evaluation; *MoF* Ministry of Finance; *PHCC* Provincial Health Coordination Committee; *WB* World Bank; *NSP* Non-State Provider; *PBF* Performance-Based Financing

**Table 3:** PBF programme actor matrix

Actors	Role	Power	Interest	Idea
Central MoPH	Policymaker	Powerful in terms of position & veto player	Interested	Supportive
World Bank	Donor/ Policymaker	Powerful in terms of having money and expertise	Very much interested	Supportive
Ministry of Finance	Policymaker	Veto player	Interested	Supportive
Third Party Organisation	Evaluator	Neutral	Neutral	Neutral
Provincial MoPH	Implementer	Powerful at the provincial level in terms position.	Publicly interested, privately not interested	publicly supportive, privately neutral
Non-state providers	Implementer	Not powerful but can influence the implementation of services	publicly interested, privately not interested	publicly supportive, privately feeling burden
Healthcare workers	Service provider	Not powerful but can influence the implementation of services	Interested	Supportive

In principle, the function of the PHOs was to serve as an arm of MoPH in achieving its provincial stewardship role. However, the role of the PHOs was restricted in every facet of PBF, including monitoring. The PBF was managed on a central basis with MoPH maintaining direct contact with the NSPs. PHOs were engaged with PBF only in two provinces where the implementation of BPHS was with the MoPH-SM.

*“The PHOs did not actively participate in the implementation of the PBF. It was obvious that they were not considered an essential actor in the design and management of the PBF” [HM, provincial level].*

The WB role in PBF programme design, financing and management was crucial. The PBF programme was designed by the WB experts given the MoPH did not have enough expertise in PBF programming during the design stage. Meanwhile, the WB maintained its crucial role in other areas. The WB was playing an operational role in appraising and supervising PBF programme activities. The PBF procurement and financial plan, the procurement process of contracting NSPs and third party, the release of funds to implementers, and hiring of staff for the PBF project all required the approval of the WB [50]. Some national and provincial managers expressed the opinion that it was the donor who made the final decisions on PBF.

*“The role of the MoPH in project design and management did not seem to be as prominent as the donor was perceived to make all important decisions” [HM, provincial level].*

Nevertheless, policymakers at MoPH disagreed with this contention and emphasised their stewardship function regarding the management and coordination of every development projects, including the PBF.

*“Overall, the MoPH relationship with the donor was either to convince or to be convinced” [PM, national level].*

The function of the third party was to verify HMIS data of the PBF programme and undertake baseline and end-line surveys to evaluate the effect of the PBF programme. Moreover, the third party had the responsibility of assessing the quality of PBF health facilities. Initially, the Johns Hopkins University (JHU) and subsequently the KIT Royal Tropical Institute assumed responsibility for this role in 2013 through a competitive process. To maintain independence, the third-party role was limited in the decision-making process, although health managers felt that this party could have taken a more active role in the design stage as well as in improving the programme implementation.

The MoF was the prime recipient of PBF funds. The MoF role was to strengthen donor coordination, to ensure the accountability and transparency of aid assistance including the PBF, and to align donor funding in accordance with the country development objectives. However, the MoF did not participate in the design and management of the PBF programme as the MoF delegated full authority to MoPH for financial management, programme choices and implementation.

*“The MoPH was regularly updating the MoF on the PBF progress. Also, the World Bank had regular meetings with the MoF. Overall, the MoF never interfered in the PBF issues” [HM, national level].*

The implementers (NSPs and MoPH-SM) assumed responsibility for implementing the PBF programme in the BPHS health facilities. Nevertheless, the NSPs function in the design of PBF was limited. On the other hand, the implementers perceived the PBF programme to be a burden because they gained no advantage while being under significant pressure to provide timely HMIS reports to MoPH and timely incentive payments to health facilities.

*“Trust me it [PBF] was a good programme but a nightmare for us (NSPs), a lot of work!” [HM, provincial level].*

Healthcare workers were the principal service providers in the BPHS health facilities. Although their role in the design of PBF was limited, and they were not involved in the policy decision-making process, most of them were satisfied with the PBF programme. They gave two reasons for this. Firstly, the PBF performance incentive was simply an extra payment to support their current standard of living. Our finding elsewhere shows that performance payments amounted almost the same level of their monthly salaries [35]. Secondly, health workers regarded performance payments as a sign of appreciation from their supervisors and a reward for efficient work.

*“Life is very expensive nowadays. The incentive I receive has changed my life. I am really happy! [HW, health facility level]*

On the other hand, although healthcare workers knew of the PBF objectives and expected outcomes, they misinterpreted the notion of allocating the health facilities into intervention and control groups. The majority of staff at control facilities were of the opinion that if they improved their performance, they could be entitled to incentive payments in the near future. National-level health managers believed that the provincial managers intentionally disseminated such messages to control facility staff to encourage them to work harder to improve the overall performance of BPHS implementation.

*“The provincial managers kept promising control health facilities to provide them incentives if they show better performance” [HM, national level].*

The implementers (NSPs, MoPH-SM) had to prepare written agreements with each health facility and define the prices of indicators and the proportion of allocation of incentives among the health facility staff. Initially, this was based on healthcare worker input and discussion. However, this was a matter of dispute in some health facilities. For instance, midwives attempted to justify the significance of their services. By contrast, other staff of health facilities, especially doctors, were of the opinion that midwives were dependent on their cooperation in order to provide services. In other cases, auxiliary staff were excluded from incentive payments, with detrimental consequences for service utilisation in some instances.

*“We noticed that our OPD [outpatient department] visits were decreasing day by day. We discovered that the guards, who were the first point of contact in the clinic, were misleading the patients. As soon as the guards were included in the PBF incentive list, the number of OPD patients increased” [HW, health facility level].*

Therefore, the managers (NSPs and MoPH-SM) subsequently defined incentive allocation schemes without the consent of healthcare workers and imposed it on some health facilities.

Some key cadres were not considered for the incentive payments, such as community health workers (CHWs) who had responsibility for the provision of basic preventive and promotive services to between 100 and 150 households and referring patients from community to health facilities.

*“CHWs are the first point of contact for patients at the community level. Frankly, they have enough influence in the community. People usually listen to what they say” [HW, health facility level].*

### PBF Implementation process

To authorize the PBF programme, a memorandum of understanding (MoU) was signed in 2008 between the MoPH and the WB [51]. A further financial agreement between the Ministry of Finance and the WB was signed in 2009 [52]. To support PBF implementation, the WB pledged 12 million US dollars grant which was utilised in six years. Negotiations between the MoPH and the WB on the management structure of PBF commenced in 2009. To furnish MoPH officials with details of the PBF such as the design and management of the programme, the WB encouraged discussions with the WB experts who had experience from the PBF in Rwanda.

In 2009, the MoPH initiated a working group to address the PBF requirement for health systems to be strengthened and to identify target provinces for the implementation of the PBF programme. The working group recognised the urgent need to strengthen the HMIS, monitoring and evaluation systems and financial management. Given that PBF required close monitoring; the working group recommended to implement PBF only in provinces where the level of security was good. Two provinces were selected as pilot sites for three months in early 2010 to identify potential administrative challenges prior to roll out [53]. As no major challenges were encountered, the PBF programme was subsequently rolled out to the remaining 9 provinces by 2011. In the initial stage, orientation sessions were also offered to BPHS implementers and provincial health officers to acquaint them with the principal features of the PBF programme.

Furthermore, the MoPH signed contracts with NSPs in nine provinces where they implemented the PBF programme in BPHS health facilities, and with the Johns Hopkins Bloomberg School of Public Health (JHU) as a third-party institution to verify the HMIS data and assess the PBF programme. Additionally, the MoPH assumed responsibility for implementing PBF in two MoPH-SM provinces [37].

In order for incentives to be paid, reported activity had to be verified by a third party. To this end, health facility HMIS data on target indicators were provided quarterly to MoPH. The verification of HMIS data occurred on a three-monthly basis between 2010 and 2013 and a six-monthly basis afterwards on a random selection of health facilities. Facility HMIS data were compared to data in facility registers. In addition, five households for each indicator were interviewed by the third party to verify that the services had been provided. In order to receive incentives, the facility validation rate had to exceed 90 per cent, and the community validation rate exceed 80 per cent. The incentive payments were weighted according to quality of care, which was assessed by a quarterly score on the national monitoring checklist (Fig. 1). In addition to facility-level incentive payments, PBF performance payments were also paid to NSPs and MoPH provincial health officers. It was anticipated that NSPs would receive 10 per cent of the performance payment paid to facilities for management purposes: this would be paid at a provincial level. The objective of this allocation was to help implementers manage the operational activity associated with the PBF. Besides, it was anticipated that provincial health officers would receive performance payments to enhance the stewardship function of the provincial MoPH associated with the PBF. Provincial health officers were paid based on the number of health facilities in provinces they monitored PBF programme quarterly, number of recorded minutes from the Provincial Health Coordination Committee (PHCC) meetings (held quarterly among actors at the provincial level) and the proportion of activities implemented from the provincial quarterly work plan. However, the allocation of management funds to implementers as well as the payments to provincial health officers was discontinued in the second year of the PBF programme. This may have occurred due to difficulties managing payments to NSPs and assessing the performance of provincial health officers [54].

The level of incentive to be paid for services at the facility-level was based on the respective burden of disease, the potential to increase coverage, the cost of service delivery in the private market, and the availability of funds. However, initially, incentives were low, but it was increased during the second year of the PBF implementation.

*“The data shows that the total amount of incentive earned by each health facility in the last three quarters is too small. Discussion with implementers has revealed that this is partly due to the unit price amount which is too small to motivate the health workers. It is agreed to revise the prices of the outputs”* [55].

The facility-level incentives were paid based on extra use of services above the baseline for the services. Therefore, the baselines for each indicator were fixed for each health facility according to the 2009 average HMIS data. It soon became apparent that the baseline had been set too high due to the inaccuracy of HMIS data in 2009. Consequently, this was amended in 2011 by applying the HMIS 2010 average data.

*“Implementing organisations expressed concern that the baseline against which performance is assessed is set too high. It is agreed to revisit the baseline”* [55].

It was anticipated that PBF performance payments would be available to implementers every six months, whereas implementers were meant to incentivise healthcare workers every three months. However, lengthy delays occurred in making payments to both implementers and healthcare workers.

*“We were told that we would receive incentives each quarter, but this was not the case. Sometimes the delays were so long that we could forget about the PBF incentives” [HW, health facility level].*

There were many reasons for the delays, including financial bureaucratic processes within the government and delaying the release of funds, implementers submitting HMIS reports late, and third-party submitting verification reports to MoPH late. In 2011, the fund delay for PBF health facilities was for three quarters. As a result, the MoPH decided to make the incentive payments to health facilities without verification of HMIS data.

*“Last year’s findings regarding third party verification showed 95 per cent accuracy of data. Therefore, the incentives should be paid on the basis of the previous year’s report to avoid further delays in performance payments.”* [53].

The verification process was found to be too resource-intensive and cumbersome. The third party faced challenges identifying households in the community from facility registers due to incorrect names and addresses. Furthermore, recall bias was a challenge with households.

*“When a monitor asked a woman whether she had visited the health facility, she was confused in her understanding of which services she had received during her visit from the health facility. In most cases, the patient cards were not available at the household level, or they contained incomplete information which made it impossible for community monitors to verify the services.” [HM, national level]*.

Some of the managers and healthcare workers argued that PBF could have worked efficiently with fully functional health facilities. Consequently, they felt it would have been better to spend some of the funding of PBF on inputs such as medicine, staff training, equipment, and supplies, all of which were needed by the BPHS health facilities.

*“We found ourselves handcuffed by the insufficient availability of pharmaceuticals, dysfunctional [medical] equipment, and lack of, particularly female healthcare workers. I wish the PBF could have helped” [HM, provincial level].*

The managers also expressed a stronger preference for the demand side-financing programme. They argued that this would have brought greater benefits as they believed that the key reason for the low utilisation of services was high transportation costs and poor road quality.

*“In extremely impoverished communities, where geographical and financial access is limited, a complementary strategy of cash vouchers allowing women to access antenatal care and facility deliveries would have resulted in a better outcome” [HM, national level].*

Table 4 presents the PBF programme lifetime timeline.

**Table 4:** PBF programme timeline

Date	Main Feature
July 2008	Afghanistan National Risk and Vulnerability Assessment report 2007/2008 released. The report highlighted that only 37 % of children received full immunisation, CPR was 15 %, 36 % ANC use, and 24 % SBA use. The cost of transportation was indicated as the main barrier to access health facilities by women and children.
September 2008	A preliminary MoU signed between MoPH and WB to adopt PBF.
April 2009	Health financing and sustainability policy and strategy developed and highlighted the need for supply and demand-side financing
October 2009	Financial agreement on PBF signed between the WB and Afghan MoF. The WB pledged 12 million US dollars grant to be used by the PBF programme.
Early 2010	PBF programme pilot started in two provinces (Panjshir and Samangan)
September 2010	PBF programme expanded to additional nine provinces (Badakhshan, Balkh, Bamyan, Jawzjan, Kandahar, Kunduz, Takhar, Parwan, Saripul)
December 2010	PBF workshop conducted to orient the PHOs and NSPs on the PBF objectives, mechanism of implementation, expected outputs and outcomes. The participants were managers from the MoPH and NSPs.
June 2011	PBF baseline survey submitted to MoPH
July 2011	PBF national workshop conducted to share the HMIS findings, discuss the unit costs of services, and find out challenges and way forward.
November 2011	PBF unit cost of services modified. PBF national workshop conducted to present HMIS updates.
February 2013	PBF workshop conducted to discuss about monitoring findings, implementation challenges, 3rd party verification results, implementation challenges and way forward. The participants were managers from the MoPH and NSPs.
Early 2016	PBF end line survey 2015 submitted to MoPH

*CPR* Contraceptive Prevalence Rate; *ANC* Antenatal Care; *SBA* Skilled Birth Attendance; *MoU* Memorandum of Understanding; *PBF* Performance-Based Financing; *WB* World Bank; *PHOs* Provincial Health Offices; *HMIS* Health Management Information System; *MoPH* Ministry of Public Health; *NSPs* Non-State Providers

## Discussion

PBF programmes are inherently political as they enforce distinct arrangements for the sharing of resources, and represents a risk or opportunity to actors as a result of changes to their roles and responsibilities and the modification of organisational processes [26]. However, despite widespread implementation of PBF programmes in LMICs, there has been minimal use of political economy analysis to shed light on why PBF is adopted, and how it is designed and implemented, including why it may not work as planned.

This study highlighted the main dynamics that influenced the adoption, design and implementation of PBF programme in Afghanistan from the lens of political economy.

### Contextual factors

 It was found that a number of contextual factors supported the adoption of PBF in Afghanistan. In general, PBF is seen as a means of achieving global policy goals, initially MDGs 4 and 5 and later UHC. A lot of countries, especially low-income and FCAS were implementing PBF [56] which supported policy uptake in Afghanistan. Besides, Afghanistan embarked on PBF based on the successful implementation of PBF in Rwanda context. Likewise, PBF was seen as an opportunity to improve the provision of healthcare services rapidly. PBF thus aligned well with donors and the Afghan government’s wish to produce fast results. Meanwhile, the strategic importance of promoting policy ideas that go with financial support is quite aligned with the interest and idea of donors in PBF. Donors are mostly concerned about achieving their results-oriented programme. Therefore, they see PBF as a suitable programme given it involves the establishment of organised, accountable, and traceable reporting system [56]. In Afghanistan, the promise of PBF financial resources came at a time when Afghanistan was encountering not only poor health indicators but also a lack of financial resources to upgrade the country’s health system. This finding is in line with other health systems performance studies that the availability of funding was a key factor influencing health policy uptake in LMICs [57, 58]. On the influence of wider political economy constraints, availability of adequate inputs such as drugs, supplies, staff training and equipment remain essential to produce health outcomes [59]. In Afghanistan’s PBF programme focused on results, while sufficient inputs were not provided to health facilities. This finding is similar to those of other studies showing that insufficient inputs in health facilities significantly influence the effectiveness of PBF programmes [59, 60]. In Burundi, the PBF programme enjoyed sufficient funds for inputs from input-based payment that led to a better performance of PBF programme [61]. In contrast, in Rwanda, the PBF programme did not prove to be pro-poor due to the insufficient inputs in health facilities to meet the needs of the poorer segment of the population [62].

### Power Dynamics

 The policy process underlying the design and implementation of the PBF programme in Afghanistan was a result of power dynamics and interactions between PBF programme actors. The exercise of power occurs not only between actors usually considered powerful, such as donors, but also actors who were influential in specific local settings such as PHOs, NSPs and health facility workers. The PBF programme established a centralised management structure to have more control on resources. Though this arrangement posed the MoPH and the donor in a strong position to manage the PBF programme through a ‘single-window system’, it compromised the notion of institutional embedding which required the engagement of all relevant units in managing the programme to prevent any drawbacks. For example, in Uganda, inattention to the role of some key actors partially led to the failure of the programme [63]. In addition, having inadequate knowledge of PBF programming, the MoPH allowed extensive external assistance in the design stage of the PBF programme which led to a flawed design such as focusing only on supply-side financing without assessing the need for a demand-side financing programme. Several surveys in Afghanistan highlighted the need for a demand-side financing programme, especially addressing the high cost of transportation to access care [64–66]. Furthermore, the donor had maintained control over the PBF programme procurement and financial decisions during the implementation stage that compromised the notion of local ownership. In this context, the PHOs and NSPs were publicly showing their interest in PBF while privately they assumed it as a burden without gaining an advantage. The findings of the present study are similar to those of other researchers, which affirm the role of donors in numerous cases with regard to setting agendas [67] as well influencing the decision-making processes with regard to health financing policies in LMICs [68]. The overriding influence of donors could lead to frustration and mistrust between donors and recipient countries, as witnessed in the context of the PBF programme in other countries [28]. In Tanzania, the PBF policy process was politicised with outside actors having considerable influence on the agenda, thus allowing minimal flexibility for the Tanzanian authorities to effectively lead the process [28]. PBF can be successful if actors take on responsibility for the programme. Kiendrebeogo and Meesen [69] suggest that all actors should assume joint ownership of a new programme as each could possess knowledge that is essential. The feeling of ownership should be engendered nationally in order that all relevant actors can value and conform to the programme. The processes of interaction with actors and the implementation approach should retain flexibility, thus providing time for the development of ownership and local capacity, and to enable integration within the health system [30]. Therefore, the country where PBF is implemented should make sure (i) to advocate for support from political individuals and institutions for the PBF programme and ensure local actors are engaged in formulating and adapting design to the local context [70]; (ii) to engage frontline healthcare workers, especially in the design process of PBF. On the basis of the Street-Level Bureaucrats model developed by Michael Lipsky [71], as frontline public workers (so-called street bureaucrats) are responsible for implementing public policies, they are capable of reshaping the policies based on their own interests and principles; hence, it is critical that their ideas are incorporated into the policies to facilitate effective implementation. For example, the involvement of community health workers in the design and implementation of PBF in Afghanistan could have improved the overall performance of the PBF programme; and (iii) to balance the influence of donors. Donors bring money that generally affords them a dominant position within policymaking processes and implementation. Nevertheless, money is not the only vehicle through which decisions can be influenced. Holding a critical position and possessing technical expertise are the two other key factors that enable actors to assume a powerful position. Hence, the MoPH could augment its ability to amalgamate its key role with technical expertise to strengthen its level of influence, and ensure programme designs are adapted to the local context [72].

### Path dependency

 The MoPH support for PBF adoption was partly linked to their past positive experience of performance-based contracting. In political economy, this is called path dependency, the notion that a new policy is shaped by the policy choices of the past [73]. However, while path dependency can influence policy choice, the capacity of an organisation in implementing a new policy is equally vital. In Thailand where the population enjoy universal health coverage, in addition to path dependency, it was the management capacity that facilitated the process of implementing related health financing reforms [74]. In Afghanistan, the health system lacked an adequate capacity to manage the PBF programme on a large scale. Thus, the PBF programme encountered implementation challenges such as delays in HMIS reports and payments, challenges in data verification, disagreement about the distribution of incentives among health facility staff, and misunderstanding of the concept of PBF in control health facilities. As demonstrated in Burundi and Rwanda, national level management capacity, especially in human resources for health, was an essential enabler to scaling up PBF programmes at the national level, whereas Kenya’s insufficient management capacity significantly affected the expansion of the PBF programme [11]. Therefore, it is highly important to ensure adaptability and responsiveness of the PBF programme design to the local context, and the availability of the local capacity to manage the implementation of RBF [75].

### Limitations 

Specific methodological weaknesses in our study also need to be acknowledged. Firstly, the interviews were conducted retrospectively. Thus, participants were asked to recollect events that happened in the past and this may have led to recall bias. To mitigate the risk of recall bias, some methodological approaches such as selecting informed participants were considered, giving the study participants enough time to think before answering the questions and using a standardised and well-structured questionnaire. Secondly, data analysis was done only by the PI. Though this could have introduced bias, the findings were triangulated with PBF documents to the extent possible. Thirdly, the study PI was working for the Ministry of Public Health in a senior position during the lifetime of the PBF programme and his opinion might have biased the study findings. On the other hand, his in-depth understanding from the local context, familiarity with the local languages, and having smooth access to senior level actors benefited this study. Fourthly, this study did not include service users (patients). Future studies could consider the inclusion of service users to understand to what extent PBF is in line with their needs. Fifthly, our case study was limited to the BPHS; the discussion on PBF could have been expanded to the Essential Package of Hospital Services (EPHS) in Afghanistan. Future PEAs could therefore include EPHS within the scope of their research to portray the picture of PBF in secondary healthcare services in Afghanistan, which may differ from primary care. Lastly, patient and the public were not involved in the design and other stages of this study. However, it is planned to disseminate the findings through publication and poster and oral presentations in conferences and public events.

## Conclusions

This study shows that successful implementation of PBF programmes needs alignment with political economy factors. In a situation in which PBF programmes are adapted according to the local context, and the interactions between actors are well managed in all stages of the policy cycle, a PBF programme can meet its objectives successfully. In Afghanistan, political economy factors played a critical role in the introduction, design and implementation of PBF programme. Future studies should focus on conducting empirical research to not only understand the multiple effects of PBF programmes on the performance of health systems but also the main political economy dynamics that influence the PBF programmes in different stages of the policy process. This will facilitate the design and implementation of an effective and flexible PBF model, adapted to the local context and owned by the country. If PBF programmes are designed around a full understanding of political economy, PBF can potentially be a powerful tool to achieve better outcomes. Further use of political economy analysis in such studies is recommended.

## Data Availability

Please contact first author to request data
